# Celiac Disease and Autoimmune Diseases in a Pediatric Population in Morocco: A Cross-Sectional Study

**DOI:** 10.7759/cureus.61468

**Published:** 2024-05-31

**Authors:** Hanane Hajaj, Aziza Elouali, Amal Hamami, Abdeladim Babakhouya, Maria Rkain

**Affiliations:** 1 Department of Pediatrics, University Hospital Mohammed VI, Faculty of Medicine and Pharmacy, Mother and Child Health Laboratory, Mohammed First University, Oujda, MAR

**Keywords:** diabetes type 1, autoimmune thyroiditis, child, autoimmune disease, gluten, celiac disease

## Abstract

Introduction: Celiac disease (CD) is defined as an autoimmune disease (AD) caused by gluten ingestion in genetically sensitive individuals. Several publications have demonstrated the increased risk of AD in patients with CD, both adults and children, which requires systematic research. Our study aimed to determine the prevalence of AD in 60 patients diagnosed with CD and to highlight risk factors that may contribute to the emergence of AD.

Materials and methods: We collected medical data from all CD patients under 16 years of age who also had AD. Our study was conducted in the Gastroenterology-Hepatology and Pediatric Nutrition Unit of the Pediatrics Department of the Mohamed VI Hospital and University Center in Oujda, Morocco, during a seven-year period between January 2017 and January 2024.

Results: We studied 60 patients with CD in our study. Eight patients (13%) had an associated AD. Their average age was eight years, with extremes varying between two and 15 years. AD was diagnosed before CD in six cases (75%), in parallel with CD in one patient (12.5%), while in only one case, it was diagnosed after CD (12.5%). All our patients had a single AD associated with CD. These ADs were mainly type 1 diabetes in seven cases and autoimmune thyroiditis in only one case. All our patients followed a gluten-free diet in addition to specific treatment for associated AD. Nevertheless, despite regular medical follow-up and targeted dietary advice for the management of CD and associated AD, three patients encountered difficulties in following the recommended diet.

Conclusion: Younger patients with CD have an increased risk of hypothyroidism and insulin-dependent diabetes. These data necessitate improved surveillance to discover these illnesses as early as possible in order to optimize management and reduce related consequences.

## Introduction

Celiac disease (CD) is an immune-mediated systemic disease caused by gluten consumption in genetically susceptible individuals. The general incidence of CD is 1.4% depending on serology and 0.7% based on biopsy findings [1]. The prevalence of autoimmune disease (AD) in CD is as high as 15% [2]. Possessing one AD increases susceptibility to another. The presence of many autoimmune disorders in the same person (autoimmune polyendocrine syndrome) has been documented in the literature [3]. Early diagnosis and a family history of autoimmunity are indicators of risk for the emergence of AD in celiac patients [4]. The effect of a gluten-free diet on the development of AD is still under debate [5]. It has been proposed to explain the association between CD and the development of AD on a shared pathogenic basis, including genetic exposure, external factors, and potentially unknown mechanisms [2-6].

The aim of this study is to estimate the prevalence of AD in patients diagnosed with CD and identify potential risk factors linked to the development of AD.

## Materials and methods

We conducted a cross-sectional study among 60 children followed up for CD in the gastroenterology-hepatology and pediatric nutrition units of the pediatrics department of the Mohamed VI Hospital and University Center in Oujda over a seven-year period from January 2017 to January 2024. The diagnosis of CD was established through clinical signs, coupled with laboratory confirmation of serum antitissue transglutaminase antibody IgA levels exceeding 10 times the upper limit of normal, along with serum IgA testing to rule out a selective IgA deficiency. The participants who were positive for serologic screening underwent an intestinal mucosal biopsy for confirmation.

The inclusion criteria used in our series were children under 16 years of age diagnosed with CD. The exclusion criteria encompassed refusal to participate, an unconfirmed CD diagnosis, and non-response to the questionnaires. A standardized questionnaire was used to assess the coexistence of AD. The data were obtained from the patient's medical files. An Excel file (Microsoft® Corp., Redmond, WA) was created to collect anamnestic, clinical, biological, radiological, and therapeutic data.

## Results

Among all enrolled patients diagnosed with MC, four were excluded either because they did not respond to the research questionnaires or because the diagnosis of MC was unclear. The mean age of the remaining 60 subjects was six years and six months, with extremes that ranged from nine months to 16 years. The most impacted age group was two to six years old. A female predominance of 53% was observed, with a sex ratio H/F of 0.85. Consanguinity was discovered in 23% of cases, with a family history of CD identified in two cases.

Out of the 60 pediatric patients diagnosed with CD, 65% were underweight, with a BMI below the fifth percentile for their age. Conversely, 35% of patients had a normal BMI. Clinical symptoms in all patients appeared gradually. However, the interval between the manifestation of the initial clinical manifestations and the diagnostic consultation ranged from one to 12 months. Additionally, 63% of the patients exhibited gastrointestinal symptoms at the time of their presentation (Table 1).

**Table 1:** Socio-demographic and clinical characteristics of our patients

Patients with confirmed CD, n = 60		
Mean age (years)	6 years	
Sociodemographics		
	Percentage	Effective (n)
Gender		
Males	47%	28
Females	53%	32
Weight centiles		
<5th centile	65%	39
>5th centile	35%	21
Height centiles		
<5th centile	47%	28
>5th centile	53%	32
Gastrointestinal symptoms		
Yes	63%	38
No	37%	22

In addition, 85% of individuals were anemic, while 50% had low ferritin levels. Vitamin D levels were below 30 ng/mL in 67% of cases (Table 2).

**Table 2:** Laboratory and histological findings of the included 60 CD patients

Patients with confirmed CD, n=60		
	Percentage	Effective (n)
Laboratory finding		
Anemia	85%	51
Low ferritin	50%	30
Low vitamin D	67%	40
Seropositivity	95%	57
Severity of histology		
Partial atrophy	32%	19
Total/subtotal atrophy	68%	41

The diagnosis of CD was established on the basis of histological and serological criteria. In addition, thyroid hormone and blood glucose levels were assessed in the majority of our patients.

**Figure 1:**
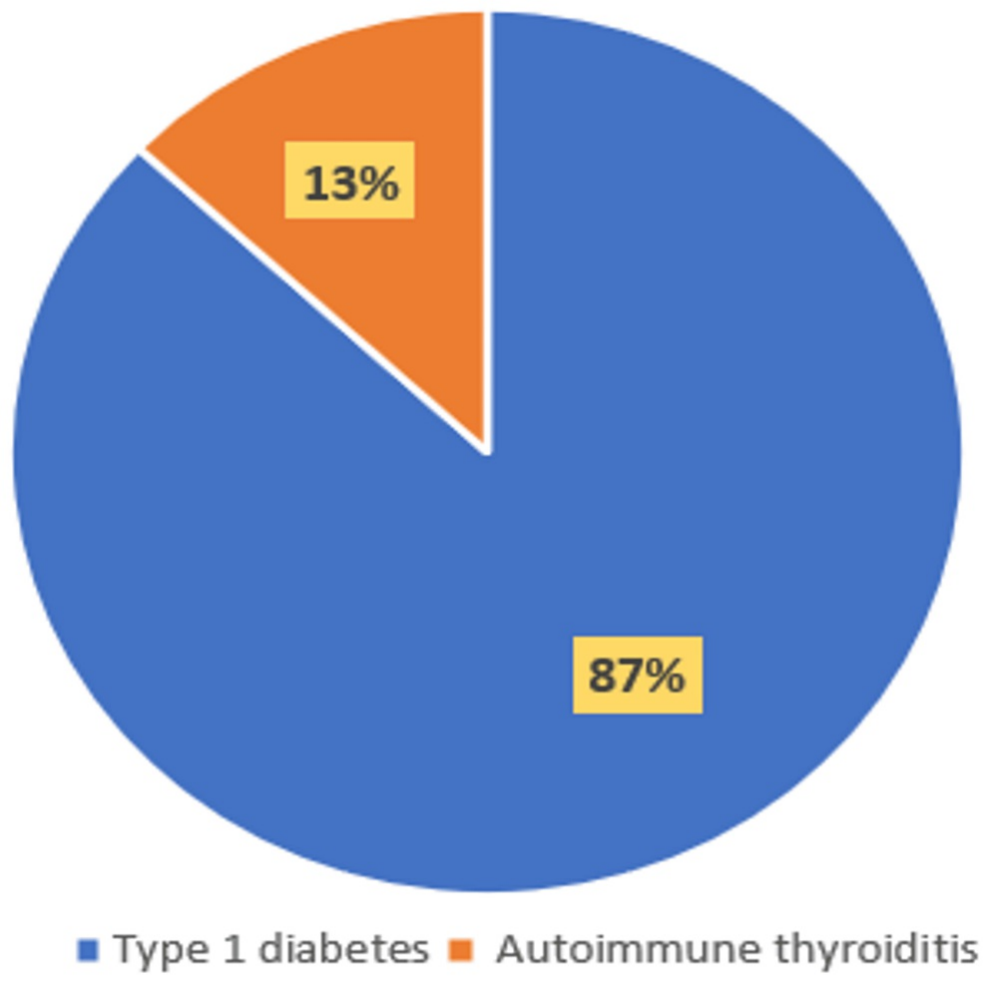
Autoimmune disease frequency among individuals diagnosed with CD in our case series

Among the patients diagnosed with CD, eight individuals (13%) had associated AD, while 52 (87%) had CD alone. Of those with both CD and AD, five were girls (63%), and three were boys (37%), giving a sex ratio (H/F) of 0.6. Consanguinity was observed in three patients (38%). The average age of these patients (MC+MA) was eight years, with extremes ranging from two to 15 years. The majority of associated AD (87%) was represented by type 1 diabetes, observed in seven cases, while autoimmune thyroiditis was present in only one case (13%) (Figure 1). Each case featured a single autoimmune disease.

In our study, it was observed that, in most cases (75%), the diagnosis of AD was made before that of CD. In one patient (12.5%), both diagnoses were made simultaneously, while in only one case (12.5%), the diagnosis of AD was made after that of CD.

All patients followed a gluten-free diet and specific treatment for associated AD. Despite regular medical follow-up and targeted dietary advice for the management of CD and associated AD, three patients encountered difficulties in following the recommended diet.

## Discussion

CD is a chronic gastrointestinal disease affecting the small intestine in genetically predisposed individuals [7]. It is associated with a variety of ADs, with around 15% of CD patients presenting with one or more ADs [8]. In our series, 13% of our CD patients had associated AD.

Individuals with only one AD have a 25% risk of acquiring further AD [9]. A shared genetic background may contribute to this association [10,11]. Younger persons with a family history of CD are susceptible to acquiring other AD [4].

As of now, based on our current understanding, there is no definitive evidence of a direct link between gluten exposure and the development of AD in patients with CD. According to an Italian multicenter study, age upon diagnosis was the sole major indicator of the appearance of AD, and the prevalence of AD in patients with CD was associated with the duration of gluten consumption [12]. Nevertheless, subsequent studies have not confirmed the protective role of gluten-free diets [13,14].

Diabetes mellitus type 1 (DMT1) is a frequent chronic disease in children, with an annual frequency of 3%-4% [15]. Regardless of race or nationality, its frequency is rising annually in every population. The frequency of CD in patients with DMT1 varies from 2.4% to 16.4% [16]. In our study, DMT1 was the most common AD in patients with CD. CD and DMT1 share common human leukocyte antigen (HLA) genotypes. Specifically, around 90% of people with DMT1 have the DQ2 or DQ8 genotypes, a significantly higher prevalence than that observed in the general population, which is around 40%. Most commonly, DMT1 is diagnosed before CD. Our study found that, in the majority of cases, DMT1 was diagnosed before CD. Adopting a gluten-free diet can alleviate growth problems in children and improve the metabolic control of diabetes. Serological screening for CD in children with DMT1 should be done at the moment of diagnosis and every one to two years subsequently [17].

The frequency of autoimmune thyroid disease rises with age in the general population. In addition, the prevalence of CD appears to be high in patients with autoimmune thyroid diseases such as Graves' disease and Hashimoto's thyroiditis. Research indicates that the frequency of CD in these individuals varies between 2.3% and 7.9% [18]. In our study, autoimmune thyroiditis was present in only one case.

According to pediatric surveys, the prevalence of CD in autoimmune hepatitis (AIH) ranges between 3.6% and 12% [19]. Mild liver abnormalities are frequently observed in individuals with CD but usually improve or disappear completely with the implementation of a balanced diet. Indeed, numerous studies have shown associations between CD and autoimmune liver diseases, including "overlap" syndrome and primary sclerosing cholangitis (PSC) [20,21]. It is important to evaluate the link between these disorders if either is found.

CD can be linked to a variety of dermatological diseases, including dermatitis herpetiformis (DH), psoriasis, alopecia areata, and vitiligo [22,23]. About 10-20% of DH patients have the classic clinical symptoms of CD, while the remaining 80-90% exhibit atypical or "silent" forms of CD [22].

CD affects 1.5%-2.8% of children with juvenile rheumatoid arthritis [24,25]. There is limited research on the prevalence of CD in other rheumatological diseases in children. There have been few studies on the favorable impact of GFD on arthritis in children.

Our study confirms the link between CD and AD, with DMT1 and autoimmune thyroiditis being the most common AD among CD-affected children. It is likely that some children included in our study have been identified with CD, but still have not been diagnosed with other AD.

Although our study provides important information, it is essential to recognize its limitations. These include its cross-sectional design and the small sample size, which prevented a complete estimation of the risk of AD associated with CD.

## Conclusions

Early detection of CD is crucial to prevent serious long-term complications such as failure to thrive, osteopenia, infertility, and malignancy. Compared to healthy people, patients with CD more frequently have concomitant AD, while patients with AD, particularly diabetes or thyroid disease, often have CD. Regular serum antibody testing, such as anti-transglutaminases IgA coupled with total IgA, is recommended for AD patients to screen for CD; however, negative serological results do not completely exclude CD.
